# First Report of *Alternaria* in the Olive Agroecosystem of NW Spain: Aerobiological Characterization and Relationship with Meteorological Factors

**DOI:** 10.1007/s00248-026-02700-x

**Published:** 2026-01-29

**Authors:** Kenia C. Sánchez Espinosa, María Fernández-González, Duarte A. Dias-Lorenzo, Francisco Javier Rodríguez-Rajo

**Affiliations:** Department of Plant Biology and Soil Sciences, Faculty of Sciences, University of Vigo, Ourense, 32004 Spain

**Keywords:** Alternaria, Airborne spores, Aeromycology, Phenology, Meteorology, Predictive models

## Abstract

*Alternaria* spp. is one of the fungal genera affecting olive cultivation, and its temporal dynamics are influenced by climatic variations occurring throughout the crop’s vegetative cycle. The aim of this study was to determine the presence of *Alternaria* spp. in an olive-growing area in northwestern Spain and to examine the relationship between its airborne concentrations and meteorological variables, in order to preliminarily predict its presence in the atmosphere during the olive tree’s phenological cycle. To achieve this, a phenological, aeromycological, and meteorological study was conducted from 2021 to 2024. *Alternaria* spp. conidia were detected in the air throughout all major phenological stages, with peak concentrations occurring mainly during fruit development. The highest percentages of spores were recorded between 11:00 and 22:00, primarily influenced by temperature and sunlight. During the maturity of fruit stages in 2023 and 2024, isolates from the *Alternaria* section *Alternaria* were identified as the cause of olive rot. A predictive model was obtained that estimates the atmospheric concentrations of this type of fungus in the study area, based on average temperature values and hours of sunshine. This study constitutes the first report of *Alternaria* spp. in an olive-growing area of northwestern Spain and provides models that preliminarily predict its presence. These models can inform growers of the pathogen’s presence in the air before visible symptoms appear, thereby reducing the likelihood of infection in susceptible plants when environmental conditions favor its development.

**Supplementary Information:**

The online version contains supplementary material available at 10.1007/s00248-026-02700-x.

## Introduction

The olive tree (*Olea europaea*) is one of the most economically significant crops worldwide [1]. In Spain, it covers 2.75 million hectares, making the country the global leader in table olive production (62% of the European Union (EU) production and 17% of worldwide production) and olive oil (70% of EU production and 45% of global production). Olive cultivation is widespread across most of the autonomous communities, with a central-southern and eastern distribution throughout the peninsula [2, 3].

Galicia, an autonomous community in northwest Spain, where the tradition of olive oil production nearly vanished due to centuries of abandoned olive groves and restrictive political and administrative measures, has gradually reemerged as a developing olive-growing region [4]. There has been an increase in the area dedicated to olive cultivation, particularly in the Miño and Sil river basins [5]. The northern and western mountain ranges shield the area from Atlantic storms, while still allowing the beneficial influence of the Mediterranean climate. This combination creates a microclimate in the Sil Valley, among the warmest and driest in Galicia [6]. However, these climatic conditions also favor the presence of fungal pathogens that damage both the aerial and subterranean organs of the olive tree during its vegetative cycle, resulting in losses in both production and quality [7].

Among the causal agents of these diseases is *Alternaria* spp., one of the most common phytopathogens in different geographical regions [8]. *Alternaria* spp., currently belongs to Pleosporaceae of Pleosporales, Dothideomycetes. Some species of this genus have been reported to be responsible for fruit rot, leaf spot and decline in cuttings in Spain, Turkey, Italy, Greece, and, Bosnia and Herzegovina. These diseases, which are emerging in olive orchards, affect the commercial value of table olives and reduce the content and quality of oil in oil-producing varieties [9–14].

The primary environmental conditions that favor the dispersal of *Alternaria* spp. conidia include temperatures ranging from 25 to 30 °C, wind speeds between 5 and 10 km/h, and relative humidity levels below 50%. These conditions promote diurnal periodicity, with peak concentrations occurring in the afternoon [15]. In Europe, several studies have examined the relationship between these variables and airborne *Alternaria* spp. concentrations, with most identifying temperature as the most influential factor [16–18]. Consequently, models have been developed to predict *Alternaria* spp. concentrations based on meteorological variables [19, 20]. However, these models are not universally applicable and must be adapted or tailored to specific geographic regions.

In Spain, aerobiological studies have focused mainly on urban areas, and reports on *Alternaria* spp. in olive crops are very limited [18, 21–25]. This lack of information prevents a proper understanding of the airborne dynamics of the fungus, its relationship with meteorological factors, and its potential impact on olive yield and quality, making it difficult to plan effective management and control strategies. Moreover, the presence of *Alternaria* spp. in the air during the olive phenological cycle could serve as an early indicator of infection risk in the fruits. For these reasons, in this study we aimed to determine the presence of *Alternaria* spp. in an olive-growing area in northwestern Spain and to analyze how spore concentrations relate to meteorological variables, with the goal of providing information useful for crop management throughout the phenological cycle.

## Materials and methods

### Location, climate, and Phenological Development of the Olive Varieties Studied

The study was conducted in an olive grove located in Ourense (NW Spain; 42°18′42″ N, 7°54′47″ W) from 2021 to 2024, from the main stage of inflorescence emergence (S5) to fruit maturity (S8). This area is 143 m above sea level and has a temperate Mediterranean climate according to the climate classification of J. Papadakis [26]. The values of the meteorological variables (maximum, minimum, and average temperatures (°C), dew point (°C), relative humidity (%), rainfall (mm), wind speed (km/h), and sun hours (h)) were obtained from the nearest (6 km) Meteorological Observation and Prediction Unit of Galicia (MeteoGalicia) weather station [27].

The determination of the phenological phases of the olive tree was carried out according to the BBCH scale adapted to olive trees by Sanz-Cortés et al. [28] (S5, inflorescence emergence; S6, flowering; S7, fruit development; S8, maturity of fruit). For this purpose, a weekly visit to the olive grove was made during the study period, except during the flowering stage, the visit occurred twice a week. Twenty-two trees, 11 of the Picual variety and 11 of the Arbequina variety, were selected for the phenological study. The beginning of each stage was considered when half of the marked trees had reached it.

### Aeromycological Study

For fungal collection, the Lanzoni VPPS 2010 spore trap was used, located in the center of the olive orchards, calibrated to aspirate 10 L of air per minute and positioned 1.70 m above ground level. Sample processing followed the methodology proposed by Galán et al. [29] and Galán et al., [30]. All conidia exhibiting the morphological characteristics described for *Alternaria* spp. by Woudenberg et al. [31] were quantified. The conidia were ovoid, obovoid, cylindrical, narrowly ellipsoid, or obclavate, with or without a beak, pale olive-brown or medium brown, smooth or warty, with transverse septa, and with or without oblique or longitudinal septa. Daily results were expressed as the number of spores per cubic meter of air (spores/m³), and annual results were expressed as the Annual Spore Integral (spore * day/m³) proposed by Galán et al. [29].

### Main Season, Daily Fluctuations, and Relative Frequency of *Alternaria*

To delimit the Main Spore Season (MSS) of *Alternaria* spp. the authors followed the method proposed by Nilsson and Person [32]. Intraday fluctuations of *Alternaria* spp. during the evaluated years were determined by selecting days without rainfall, on which spore concentrations exceeded the mean airborne spore concentration during the MSS in each year. These selected days were used to calculate the mean spore count every 2 h, and the data were expressed as percentages. Additionally, the relative frequency (RF) of spore occurrence in the air was calculated for each phenological stage using the formula: RF = (Number of days on which spores were detected during the stage/Total number of days in the stage) * 100.

### Collection, disinfection, and Isolation of Olive Fungi

In 2023 and 2024, olives with rot lesions were collected during the fruit maturity stage (S8) (Fig. S1). These were washed with running tap water, and superficially disinfected by immersing them in the following series of solutions: Sterile H_2_O for 60 s, 70% ethanol for 2 min, 0.5% sodium hypochlorite for 2 min, 70% ethanol for 1 min, and a final rinse in sterile H_2_O three times. About 100 µl of the final rinse water was inoculated onto Agar Potato Dextrose (PDA) to check surface disinfection. They were blotted dry with sterile filter paper, placed on the surface of a water agar plate (1.5%), and incubated at 25 ± 2 °C. The agar-water medium lacks nutrients and acts solely as a physical support, allowing verification that any fungal growth observed originates from inside the plant tissue rather than from surface contaminants. Plates were checked daily for any fungal growth. Isolates grown on water agar were inoculated onto fresh PDA plates and maintained at 4 °C on PDA agar wedge until morphological identification according to Ellis [33] and Woudenberg et al. [31].

### Statistical Analysis

#### Data Normality Assessment and Analysis

First, an analysis of the data distribution was performed to assess the normality of the meteorological variables and *Alternaria* spp. concentrations in the air. For visual analysis, histograms were generated for each variable using the ggplot2 package, and the Shapiro-Wilk test was applied for a formal statistical assessment of normality (*p* < 0.05).

#### Rank Correlation and Principal Component Analysis

The relationship between air conidial concentration during the MSS, by year, and for the entire study period with the meteorological variables was determined through Spearman’s rank correlation coefficient (*p* < 0.05). The correlation was represented graphically using the corrplot package. For these same time periods, a principal component analysis (PCA) was performed to evaluate the meteorological influence of all variables on conidia concentrations. The *prcomp*() function was used for this analysis. The interpretation of the results was based on the factorial plane of the variables. This graph allowed the relationship and direction of the vectors of the variables to be visualized in the two-dimensional space defined by Principal Components 1 and 2.

#### Predictive Modeling of Spore Concentrations in the Air

##### Data and Preprocessing

Airborne concentrations of *Alternaria* spp. were analyzed in conjunction with meteorological variables. The data were thoroughly explored and preprocessed for modeling, applying necessary transformations to meet statistical assumptions and to appropriately handle zero values and skewed distributions.

##### Variable Selection and Multicollinearity Assessment

Multicollinearity was evaluated using the Variance Inflation Factor (VIF) via the *vif*() and *check_VIF*() functions from the car and performance R packages, respectively. In multiple linear regression models, all meteorological variables were initially included, followed by automatic predictor selection based on the Akaike Information Criterion (AIC), with non-significant variables (*p* > 0.05) removed iteratively. For the GLMMs, variable selection was conducted manually, guided by prior exploratory analyses and the biological plausibility of meteorological effects.

##### Multiple Linear Regression (Exploratory Analysis)

Multiple linear regression models were fitted to assess the overall relationships between meteorological variables and *Alternaria* spp. concentrations. Two model variants were considered: one using the original values, and another applying logarithmic transformations to spores concentrations (x + 1). This exploratory analysis facilitated the identification of relevant predictors. Model fit and predictive performance were evaluated using R² and Root Mean Square Error (RMSE).

##### Generalized Linear Mixed Models (GLMM, Main Analysis)

To quantify the influence of meteorological variables while accounting for hierarchical effects, GLMMs with a Tweedie distribution and a log link function were fitted, appropriate for semi-continuous data including zeros. Year was included as a fixed effect, and crop phenology as a random effect. The final model was selected based on information criteria (AIC and BIC - Bayesian Information Criteria) and the biological plausibility of included variables. Predictive performance was assessed via validation R² and RMSE. Differences between years were tested for significance using Tukey’s HSD (*glht*() from the multcomp R package). The statistical software package used was R version 4.5.1.

## Results

### Temporal Dynamics of *Alternaria* in the Air and its Detection in Olive Fruits

**Fig. 1:**
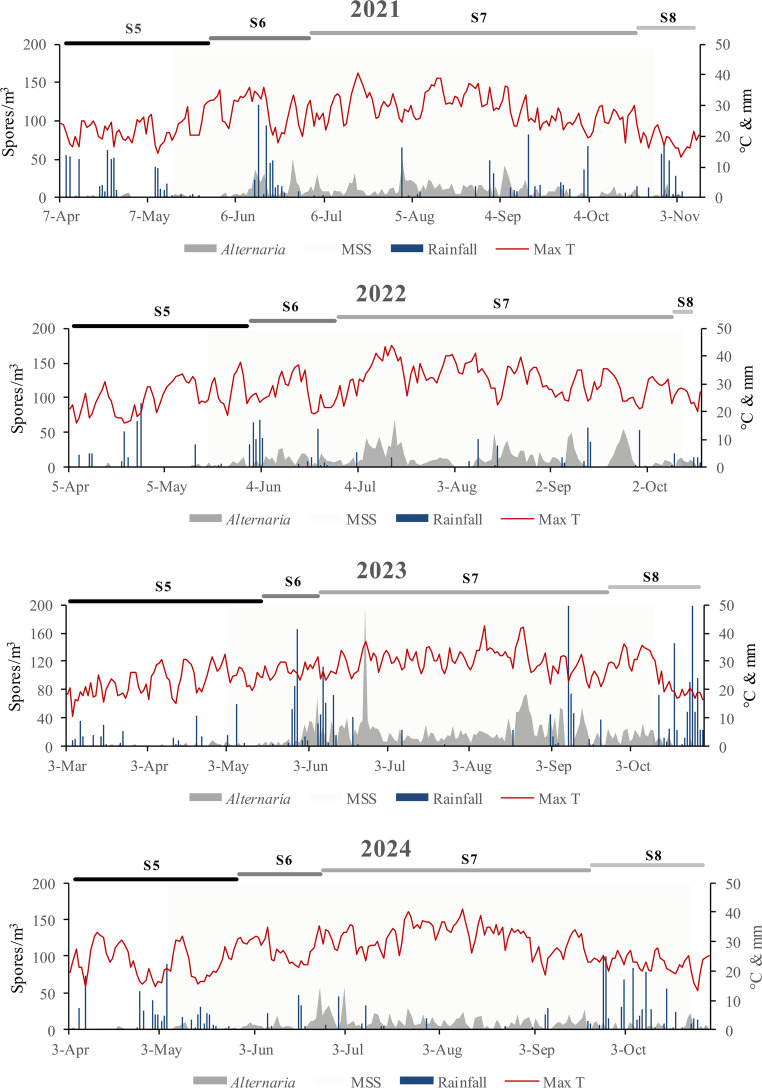
Temporal dynamics of *Alternaria* spp. concentrations in the air, maximum temperature and accumulated rainfall during the main phenological stages of olive orchards. S5, inflorescence emergence; S6, flowering; S7, fruit development; S8, maturity of fruit; MSS, Main Spore Season; Max T (°C), mean temperature.

To contextualize the aerial dynamics of *Alternaria* spp. during crop development, the phenological stages of the olive trees were recorded. The duration of these stages varied across the four years of the study (Fig. 1). The longest stage was fruit development (S7), which lasted the most days in 2021. The inflorescence emergence (S5) was the second longest in all years, with the longest duration recorded in 2023. The flowering stage (S6) lasted longer in 2021 but was shorter in 2023, when it began earlier. S8 had a shorter duration in 2022 and started later in 2021.

*Alternaria* spp. conidia were identified in the air with different types of morphology (Fig. S2). The MSS was between the months of May to October, coinciding with practically the entire evaluated vegetative cycle of the olive tree (Fig. 1). The concentration peaks were generally detected during S7, with its highest value on June 24, 2023 (194 spores/m^3^), coinciding with the year in which the highest concentrations were recorded (3146 spore * day/m^3^).

**Fig. 2:**
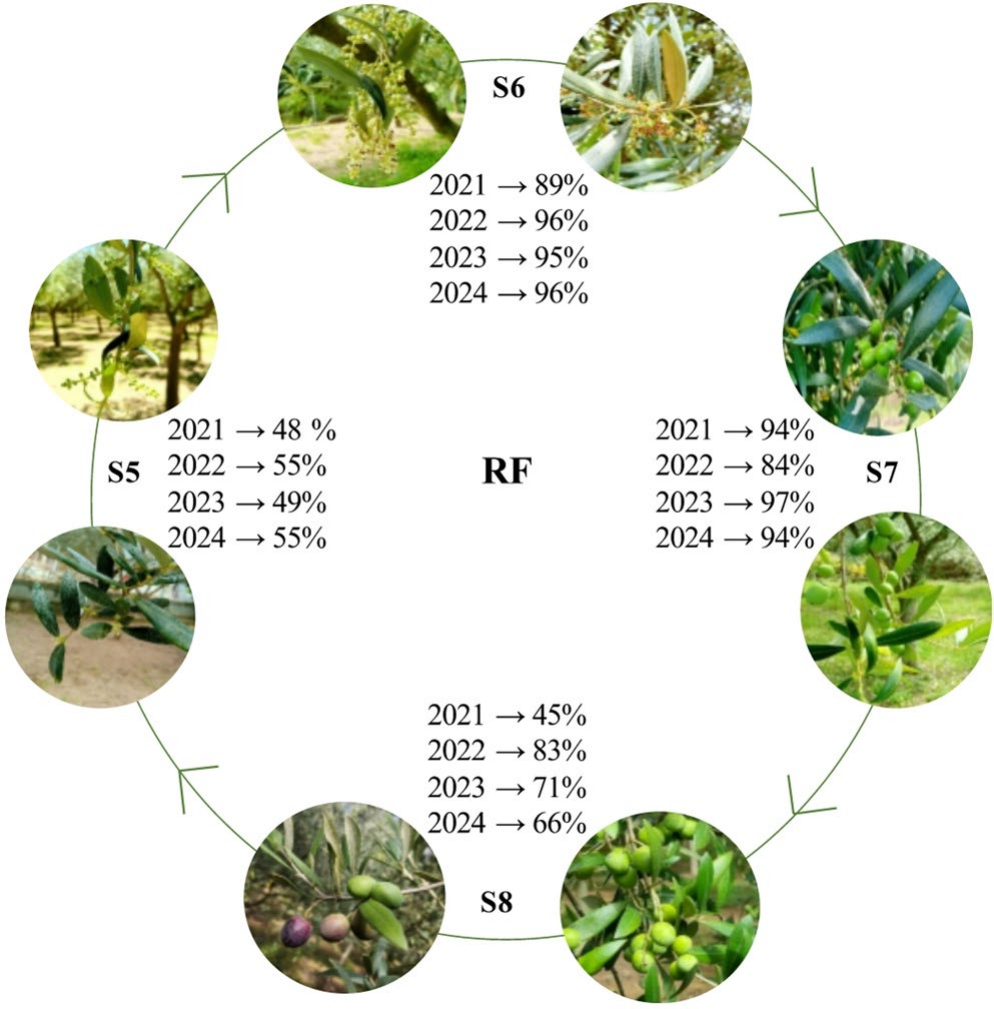
Relative frequency (RF) of detection of *Alternaria* spp. in the air at the main phenological stages. S5, inflorescence emergence; S6, flowering; S7, fruit development; S8, maturity of fruit.

**Fig. 3:**
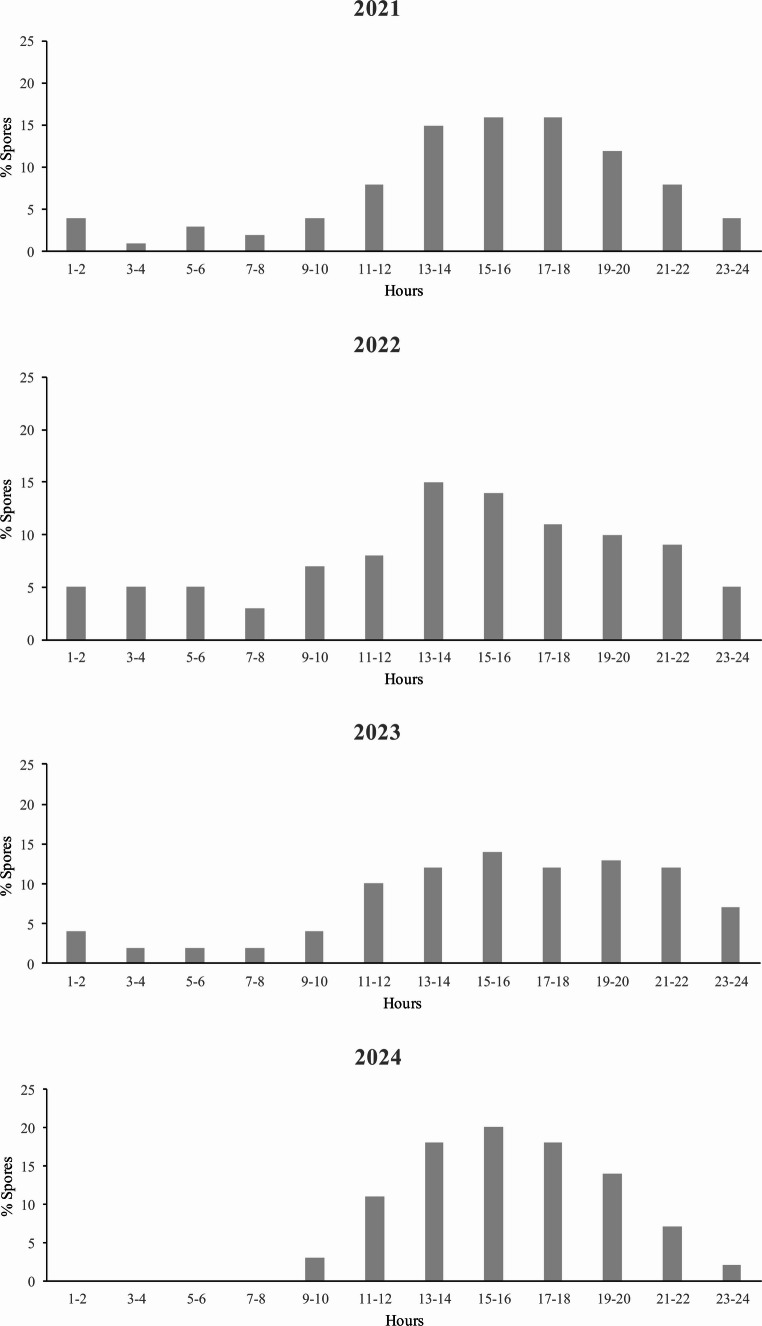
*Alternaria* spp. intradiurnal patterns during the study period (2021–2024).

The RF of its detection in S6 and S7 was higher than 84% (Fig. 2). In S5 the frequency of occurrence varied from 48 to 55% and in S8 from 45 to 83%. *Alternaria* spp. had a diurnal dispersal pattern and predominated in the air from 11 to 22 h. The highest percentages of spores were between 13 and 14 h, 15–16 h and 17–18 h (Fig. 3).

**Fig. 4:**
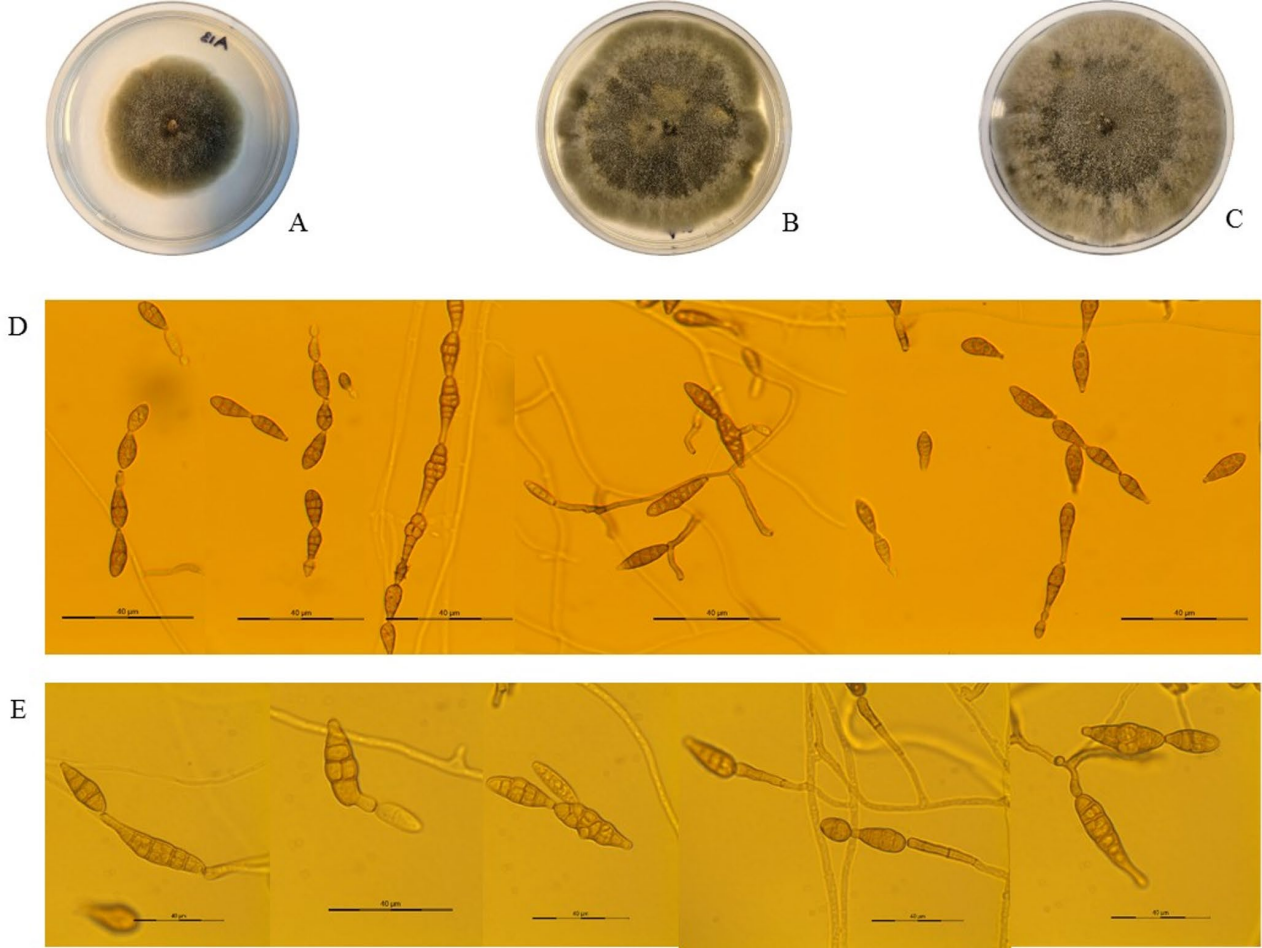
Colony morphology of *Alternaria* section *Alternaria* (A13) in PDA after 7 (**A**), 10 (**B**) and 14 (**C**) days of incubation. Photomicrograph of conidia in microcultures after 5 days of incubation on PDA at 200x (**D**) and 400x (**E**).

On the other hand, during the S8 season in 2023 and 2024, isolates of *Alternaria* section *Alternaria* were identified in olives with rot of the Arbequina variety. The effectiveness of the surface sterilization process was confirmed by the absence of microbial growth on the control plates inoculated with the final rinse water. Fungal growth was observed exclusively from internal tissues placed on water agar, confirming that the recovered isolates originated from within the fruit rather than from surface contaminants. The colonies grown on PDA, after 7 and 14 days, were olive-black and effuse, with part of the mycelium immersed in the substrate. The conidiophores were primary, straight or curved, ranging from short to long, and either simple or branched. In microcultures, chains of up to ten conidia, sometimes branched, were observed. The conidia were primarily obclavate or ellipsoid, with up to seven transverse septa, slightly constricted near some septa, and with few longitudinal septa. Conidial dimensions ranged from 9.00 to 45.27 μm in length and from 2.83 to 19.32 μm in width. The body of some conidia gradually tapered into a conical or cylindrical beak, sometimes up to one-third the length of the conidium, and pale (Fig. 4).

### Relationship between *Alternaria* spp. Spores and Meteorological Variables

Average temperatures during the study period ranged from 5.50 to 31 °C, maximum temperatures from 40 to 44 °C, and minimum temperatures from − 3.60 to 22.20 °C. The highest rainfall (52.80 mm), humidity percentages (99%), dew point, and wind speed (10.60 km/h) were recorded in 2023. The hours of sunshine had the same maximums in all years (Table S1).

**Fig. 5:**
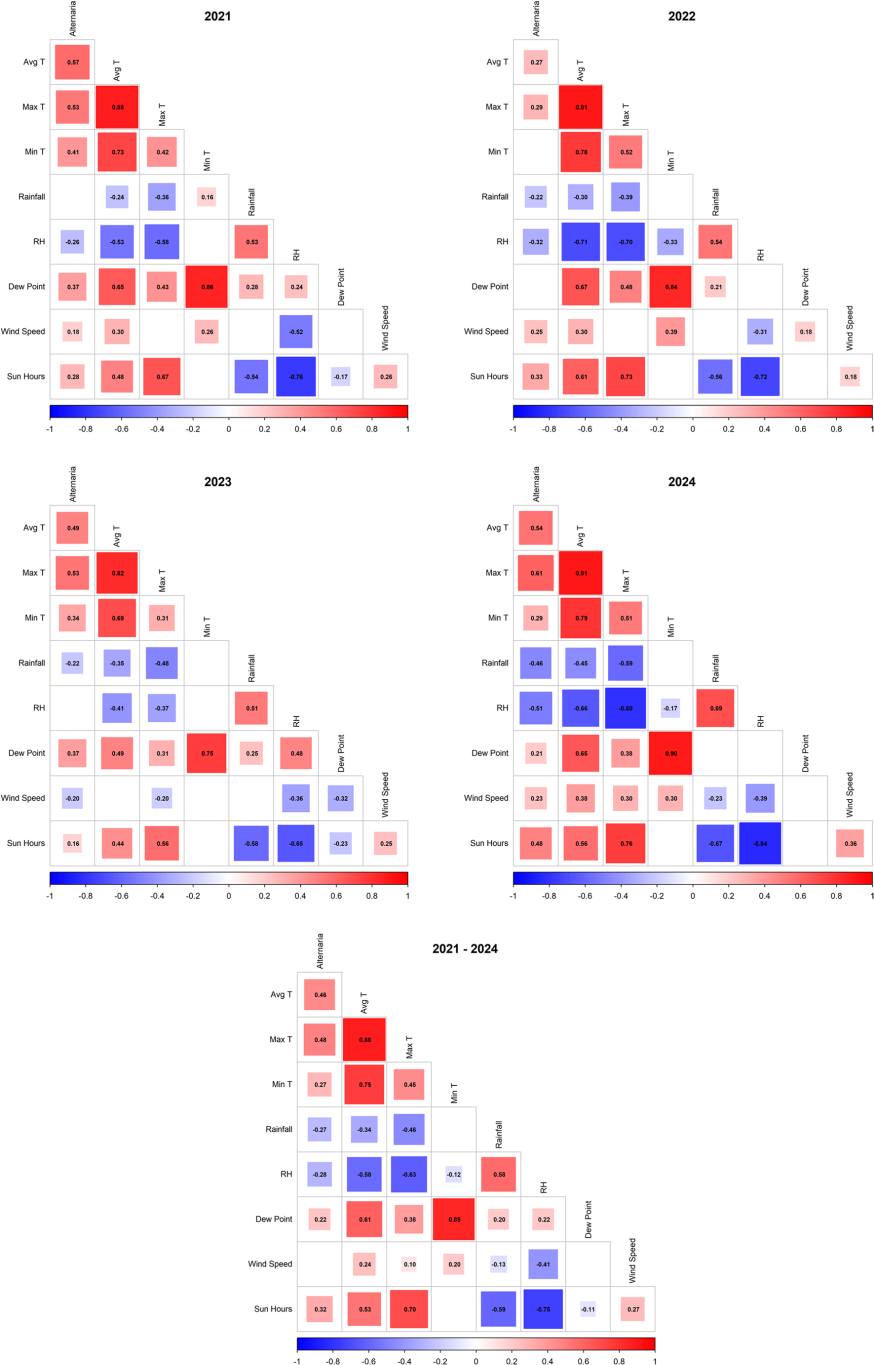
Correlograms between daily *Alternaria *spp. concentrations during the MSS of 2021, 2022, 2023, 2024, and 2021 - 2024 and meteorological variables, by Spearman's correlation method. The matrix shows Spearman's correlation coefficients between variables, with colors ranging from blue (negative correlation) to red (positive correlation). Statistically insignificant coefficients (p > 0.05) are left blank. The meteorological variables were average temperature (Avg T, °C), maximum temperature (Max T, °C), minimum temperature (Min T, °C), rainfall (mm), relative humidity (RH, %), dew point (°C), wind speed (km/h) and sun hours (h).

The Shapiro-Wilk test and histograms performed for meteorological variables and *Alternaria* spp. concentrations in the air showed that, except for the average temperature, the rest did not meet the assumptions of normality (Table S2, Fig. S3). Therefore, Spearman’s nonparametric rank correlation method was used for the bivariate analysis between these variables. This analysis revealed that the main variables positively correlated with the presence of spores in each year and during the MSS were the average and maximum temperatures, as well as the hours of sunshine (Fig. 5, Fig. S4). The dew point and minimum temperature also showed a positive correlation, except during the MSS of 2022. Wind speed exhibited a similar relationship, except in 2023 (MSS 2023, total MSS 2023, and 2023), where the correlation was inverse. Rainfall and relative humidity were negatively associated with *Alternaria* spp. concentrations in all years and during most of the MSS.

**Fig. 6:**
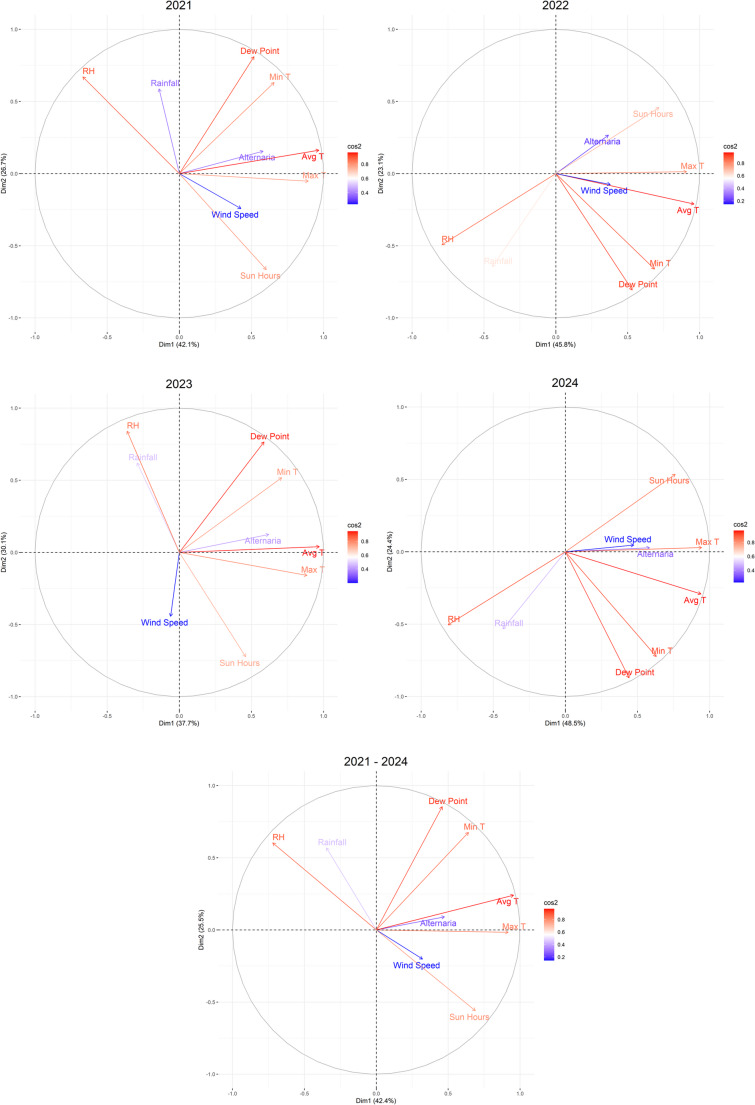
Principal Component Analysis of the MSS, 2021, 2022, 2023, 2024 and 2021 – 2024. Principal Component one (Dim1) and Principal Component two (Dim2). The meteorological variables were average temperature (Avg T, °C), maximum temperature (Max T, °C), minimum temperature (Min T, °C), rainfall (mm), relative humidity (RH, %), dew point (°C), wind speed (km/h) and sun hours (h).

The PCA results indicated that the first two principal components captured a substantial proportion of the total variability in the data (> 65%), which justifies the interpretation of the relationships in the two-dimensional factorial plane (Fig. 6, Fig. S5). In the PCA factorial plane for both MSS and full years, *Alternaria* spp. concentrations were projected in directions consistently similar to those of maximum and mean temperature and sunshine hours, indicating that these variables tend to covary in the multivariate context of the study. Relative humidity and precipitation were projected in directions opposite to those of *Alternaria* spp. concentrations, suggesting an inverse relationship in the multivariate context. Wind speed shows weak relationships. Its low loadings indicate a small contribution to the first two principal components. Regarding minimum temperature and dew point, there were differences between the analysis including the complete years and only the MSS. The analyses of the complete years show projections similar to *Alternaria* spp., while in the MSS the relationship becomes opposite or perpendicular.

The exploratory analysis developed through multiple linear regression yielded two models that explain variations in *Alternaria* spp. concentrations in the air based on meteorological variables. Both models obtained significant values in the F-test, indicating that they are effective in predicting spore concentrations and that the explained variation is unlikely to be due to chance. Model 1, which uses the total raw data, showed that the average temperature explains 19.9% of the variability in spore concentrations. Model 2, in which the fungus concentration data were logarithmically transformed, indicated that the maximum temperature and dew point together explained 36.5% of the variability (Table 1). In both models, higher temperatures were associated with increased concentrations of *Alternaria* spp., while in model 2, higher dew point values also corresponded to increased concentrations when the maximum temperature remained constant. All predictive variables included in the models were statistically significant, and there was no multicollinearity among the independent variables.

**Table 1:** Regression parameters of prediction models of *Alternaria* spp. Concentrations in air

	*R*^2^ adjusted		RMSE ^a^	Significance F-statistic	
Model 1	0.199		10.202	< 2.2e-16	
Model 2	0.365		14.377	< 2.2e-16	
		Coefficients		t - value	Sig.
		Estimate	Std. error		
Model 1	Intercept	−16.447	2.006	−8.199	**1.18e-15**
	Avg T ^b^	1.360	0.103	13.179	**< 2e-16**
Model 2	Intercept	−1.662	0.172	−9.609	**< 2e-16**
	Max T ^c^	0.092	0.007	13.045	**< 2e-16**
	DP ^d^	0.069	0.011	6.021	**2.83e-09**
Equation					
Model 1	*Alternaria* = −16.447 + 1.360 Avg T				
Model 2	Log(*Alternaria* + 1) = −1.662 + 0.092 Max T + 0.069 DP (VIF ^e^ < 2)				

^a^ Root Mean Square Error ^b^ average temperature, ^c^ maximum temperature, ^d^ dew point, ^e^ Variance Inflation Factor. Significance level in bold type (*p* < 0.001)

Taking into account the results obtained from previous statistical analyses, multiple GLMM models were constructed to evaluate the effect of the year and meteorological variables on *Alternaria* spp. concentrations, considering the phenological stages of the crop as a random effect (Table S3). Regardless of the model evaluated, the year 2023 showed significantly higher concentrations than the other years. The models that included only one meteorological variable (Model AT, Model MT, Model MiT, Model R, Model RH, Model DP, Model WS, Model SH) showed that, except for wind speed, all significantly influence the presence of spores, as did the results obtained in Spearman’s correlations and PCA.

The final model selected, called ATSH, included year, average temperature, and hours of sunshine as fixed effects, and the phenological stages of the crop as a random effect (Table 2). This model had the lowest AIC (3555.4) and BIC (3595.1) values, indicating the best balance between fit and parsimony. The results indicate that the year 2023 had a significant positive effect on the concentration of *Alternaria* spp. compared to 2021 (*p* < 0.001). The post hoc analysis confirmed these significant differences with the other years and between 2024 and 2022. Both average temperature and sunshine hours showed positive and highly significant effects, suggesting that each 1 °C increase in average temperature is associated with an expected 9% increase in *Alternaria* spp. concentration, and each additional hour of sunshine is associated with a 5% increase. The random effect of phenological stages had a variance of 0.494 and a standard deviation of 0.703, indicating that differences between these stages contribute moderately to the total variability of *Alternaria* spp. concentrations. The VIF values for the continuous variables (average temperature and hours of sunshine) were all less than 2.5, indicating no multicollinearity issues. The RMSE of the model on test data was 9.92, and the validation R² was 0.428, reflecting a reasonable model fit and moderate predictive power.

**Table 2:** ATSH GLMM of *Alternaria* spp.: Coefficients, pairwise comparisons, and model fit metrics as a function of year, average temperature (Avg T, °C), and sun hours (h), with phenology as a random effect.

	Estimate	Std. error	z - value	Pr(>|z|)
Intercept	−0.471	0.415	−1.136	0.256
Year 2022	0.140	0.110	1.270	0.204
Year 2023	0.466	0.101	4.605	4.12E-06 ***
Year 2024	−0.175	0.115	−1.520	0.128
Avg T	0.090	0.013	6.786	1.15e-11 ***
Sun Hours	0.050	0.013	3.867	0.000 ***
Multiple Comparisons of Means				
2022 − 2021	0.140	0.110	1.270	0.581
2023 − 2021	0.466	0.101	4.605	< 0.001 ***
2024 − 2021	−0.175	0.115	−1.520	0.424
2023 − 2022	0.327	0.100	3.270	0.006 **
2024 − 2022	−0.315	0.112	−2.822	0.024 *
2024 − 2023	−0.642	0.104	−6.180	< 0.001 ***
	Variance	Std.Dev.		
Phenology	0.494	0.703		
AIC^a^	3555.4		BIC^b^	3595.1
RMSE^c^	9.92		R^2^	0.428
Equation				
E[*Alternaria*_*ij*_​] = exp(− 0.471 + 0.140 ⋅*D*_2022_ ​+ 0.466 ⋅ *D*_2023_ ​− 0.175 ⋅ *D*_2024_​ + 0.090AvgT_ij_ ​+ 0.050SunHours_ij_ ​+ u_Phenology_) (VIF ^d^ < 2.5)				

^a^ Akaike Information Criteria, ^b^ Bayesian Information Criteria, ^c^ Root Mean Square Error. ^d^ Variance Inflation Factor. *D*_YYYY_ are year dummy variables (1 if it corresponds to the year, 0 otherwise), and u_Phenology_ is the random effect of the phenological stages. Significance codes: 0 ‘***’ 0.001 ‘**’ 0.01 ‘*’

## Discussion

Aeromycological studies in agricultural areas are valuable tools for crop protection. Monitoring airborne spores helps to understand spore dispersal, fungal epidemiology, and the implementation of appropriate phytosanitary measures [34, 35]. In this study, we determined for the first time the temporal variation of *Alternaria* spp., an olive phytopathogen, in an olive-growing region of northwestern Spain. We also preliminarily predicted its airborne concentrations using meteorological variables and identified *Alternaria* section *Alternaria* as a causal agent of olive rot.

The count of *Alternaria* spp. conidia reveals significant morphological diversity within this cultivar concerning the genus, which makes it difficult to identify the species of conidia based solely on the collection method used. In fact, Pyrri et al. [36] conducted a comparative study using air samples collected through the Hirst method and culture, which revealed differences in the morphological patterns of *Alternaria alternata*.

The determined MSS (May - October) aligns with the findings reported by Sánchez et al. [37] for the Galicia region and with studies from other cities in the Iberian Peninsula [23]. Maximum concentrations and the highest frequency of occurrence primarily occurred during the summer months. These results, observed during fruit development and maturity of fruit, suggest a higher likelihood of *Alternaria* spp. leaf spot and fruit rot, as optimal temperatures for symptom development also prevail [15].

Indeed, the isolates of *Alternaria* section *Alternaria* obtained from olives with lesions were collected during fruit maturity in 2023 and 2024. During fruit maturity, there is a higher likelihood of isolating this fungus from olives than during the earlier stages of fruit development, which take place in the summer months. In this season, the olive tree responds to the water stress caused by summer drought by increasing cuticle thickness, reducing stomatal conductance, and decreasing sap flow [38–40]. This reduces the points of entry and the availability of nutrients for the pathogen, thus hindering its colonization. Due to logistical constraints, sampling was not carried out in 2021 and 2022, which limited the assessment of the pathogen’s impact during those seasons and restricted the temporal scope of the study. Nonetheless, the results obtained provide relevant evidence on the occurrence and etiology of the observed damage, constituting a solid basis for future follow-up studies. The detection of species from this section as olive tree phytopathogens has also been reported in southern Spain in this same olive variety, but this is the first record in the northwest region [10]. Its occurrence has also been reported in Turkey, Greece, Pakistan, Italy, and Bosnia and Herzegovina [11, 13, 14, 41–43]. Its identification is particularly concerning, as this species may produce mycotoxins such as tenuazonic acid (TA), alternariol (AOH), alternariol methyl ether (AME), altenuene (ALT), altertoxin I (ATX-I), and altertoxin II (ATX-II), highlighting its potential significance for crop health and food safety [44].

The maximum values observed during daylight hours align with the characteristics of these dry conidia. Their development, sporulation, and dispersal typically occur around midday and in the afternoon, when higher temperatures prevail, more sunshine is present, and humidity levels are at their lowest [34]. Rodríguez-Rajo et al. [45] previously reported an increase in *Alternaria* spp. concentrations in the late afternoon (19:00–22:00) in the northwest of the Iberian Peninsula. Similarly, in the southern regions, maximum concentrations are observed between 13:00–21:00 and 17:00–20:00 [24, 46].

The combination of Spearman’s rank correlation coefficient and PCA statistical analyses provided a robust and consistent view of the dynamics of *Alternaria* spp. in relation to meteorological variables. Maximum and average temperature and hours of sunshine are the variables that most promote the presence of this phytopathogen, while relative humidity and precipitation have a negative effect. The relationship between temperature, hours of sunshine, relative humidity, and precipitation has been documented by several authors in various geographical regions [20, 47–55], and is consistent with the physiological characteristics of this genus, which produces dry conidia, as explained above. Both analyses identify a crucial inconsistency in the relationship between minimum temperature and dew point, highlighting the importance of the temporal scale of the analysis. The analysis of complete years showed a generally positive relationship, which is consistent with the results obtained by Meno et al. [56] in the northeast of the Iberian Peninsula, by Olsen et al. [57] in Copenhagen, and by Jetschni et al. [58] in Sydney. However, during the MSS, this relationship was reversed or became null. This finding suggests that, although the overall annual trend indicates that heat is favorable, at the peak of the sporulation season, minimum temperature and dew point may not be major limiting factors, and spore dynamics are more influenced by other environmental or biological factors. Wind speed showed a variable and very weak effect on *Alternaria* spp. concentrations, so it was not possible to establish a clear relationship as to whether wind gusts contributed to the release of conidia or to their dilution [24, 59–61].

Given that climate influences the concentration of *Alternaria* spp. spores in the air, we developed linear regression models and generalized linear mixed models (GLMM) to clearly identify the meteorological factors that most consistently influence atmospheric concentrations of *Alternaria* spp. It was decided to use complete annual data in order to ensure greater statistical robustness and capture the overall trend of meteorological influence.

Model 2 obtained by multiple linear regression showed that maximum temperature and dew point were the key factors most influencing *Alternaria* concentrations in the air. However, the GLMM that included these variables (Model MTDP) indicated that maximum temperature did have a significant positive effect but that dew point did not influence this variability. Although this model was not selected as the best model, it showed few differences in terms of AIC/BIC with the selected ATSH model, which included average temperature and hours of sunshine, highlighting the effect of warm temperatures on the dynamics of *Alternaria* spp., as mentioned above [52, 54, 62].

The final ATSH model performed adequately when integrating key meteorological variables and the random effect of phenological stages. Its moderate predictive capacity, together with low VIF values and favorable AIC/BIC metrics, indicates that the model captures a substantial portion of the variation without overfitting. However, it also shows that other factors not included in this study, such as microclimatic factors, orchard management, and local dispersal processes, could contribute to the remaining variability [57]. These factors could also explain the marked increase in concentrations during 2023, which was consistent across all models. Despite these limitations, the model provides a useful preliminary tool for predicting periods of increased risk. Similar meteorological predictors have been identified in studies conducted in the United States, Australia, and Europe [19, 21, 51, 59, 63].

Taken together, these results reinforce the importance of considering simple and easily monitored meteorological variables to anticipate increases in *Alternaria* spp. in crops susceptible to this phytopathogen. The significant influence of temperature and hours of sunshine suggests that warmer climatic scenarios with higher radiation, as expected in the future due to climate change, could increase the presence of these spores.

## Conclusions

This study represents a significant advancement in aeromycological research related to olive cultivation in northwestern Spain, offering the first evidence of how meteorological factors influence the temporal variation of *Alternaria* spp. in this agricultural context. The identification of *Alternaria* section *Alternaria* as a causal agent of olive rot highlights the critical role of aerobiological monitoring in crop management. These findings not only deepen our understanding of the pathogen’s epidemiology but also contribute to the development of preliminary predictive models that could help anticipate infection risks and refine phytosanitary strategies in olive orchards.

## Supplementary Information

Below is the link to the electronic supplementary material.

Supplementary Material 1 (DOCX 4.19 MB)

## Data Availability

Data will be made available on request.
