# Supplementary material for: First Report of Alternaria in the Olive Agroecosystem of NW Spain: Aerobiological Characterization and Relationship with Meteorological Factors
Source: Microb Ecol. 2026 Jan 29;89(1):50. doi: 10.1007/s00248-026-02700-x (PMC12904890; doi:10.1007/s00248-026-02700-x)
Supplement: Supplementary file 1 — Supplementary Material 1 (DOCX 4.19 MB) [file 248_2026_2700_MOESM1_ESM.docx]

**Fig. S1**. Olive damaged by rot during the fruit maturity stage.

**
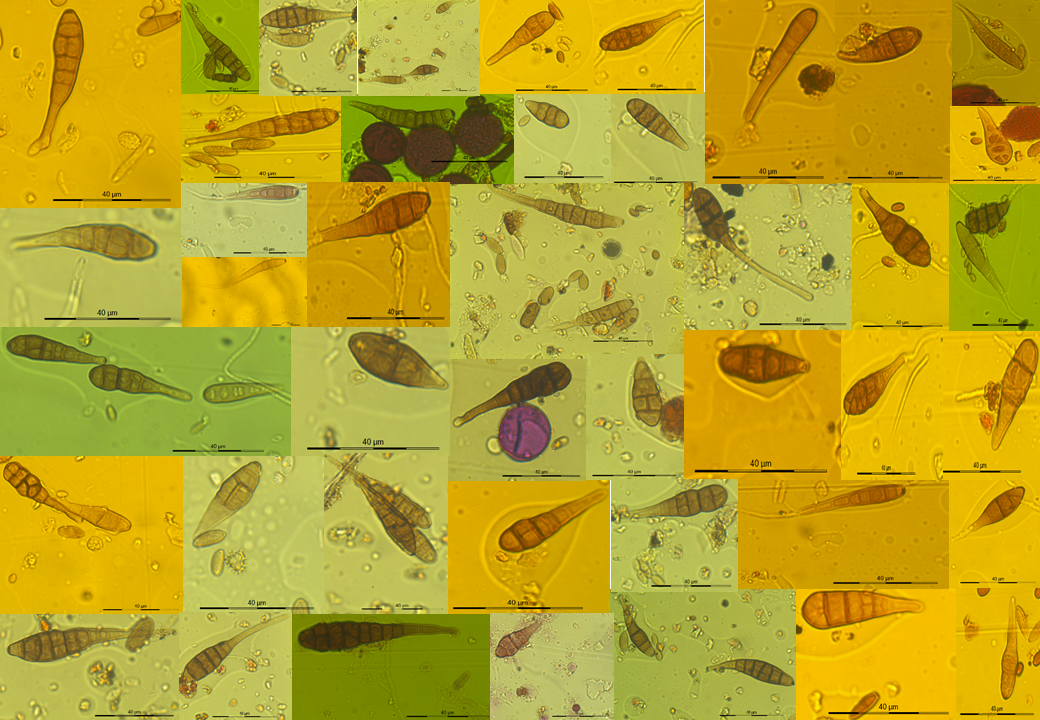
**

**Fig. S2**. Photomicrographs of *Alternaria* spp. spores identified during the study period in the aerobiological preparations obtained from the Lanzoni VPPS 2010 spore trap.

**Fig. S3**. Histograms of *Alternaria* spp. and meteorological variables (Average temperature (Avg T, °C), maximum temperature (Max T, °C), minimum temperature (Min T, °C), rainfall (mm), relative humidity (RH, %), dew point (°C), wind speed (km/h) and sun hours (h)), showing the distribution of their values.

**Fig. S4.** Correlograms between daily *Alternaria* spp. concentrations in 2021, 2022, 2023, 2024, and 2021 - 2024 and meteorological variables, by Spearman's correlation method. The matrix shows Spearman's correlation coefficients between variables, with colors ranging from blue (negative correlation) to red (positive correlation). Statistically insignificant coefficients (p > 0.05) are left blank. The meteorological variables were average temperature (Avg T, °C), maximum temperature (Max T, °C), minimum temperature (Min T, °C), rainfall (mm), relative humidity (RH, %), dew point (°C), wind speed (km/h) and sun hours (h).

**Fig. S5.** Principal Component Analysis of the 2021 (a), 2022 (b), 2023 (c), 2024 (d), and 2021 – 2024 (e). Principal Component one (Dim1) and Principal Component two (Dim2). The meteorological variables were average temperature (Avg T, °C), maximum temperature (Max T, °C), minimum temperature (Min T, °C), rainfall (mm), relative humidity (RH, %), dew point (°C), wind speed (km/h) and sun hours (h).

**Table S1**. Average, maximum and minimum values of the meteorological variables recorded. Average temperature (Avg T, °C), maximum temperature (Max T, °C), minimum temperature (Min T, °C), rainfall (mm), relative humidity (RH, %), dew point (°C), wind speed (km/h) and sun hours (h).

|  |  | **Avg T** | **Max T** | **Min T** | **Rainfall** | **RH** | **Dew Point** | **Wind Speed** | **Sun Hours** |
| --- | --- | --- | --- | --- | --- | --- | --- | --- | --- |
| **2021** | Average | 17.82 | 26.29 | 11.35 | 1.89 | 70.10 | 11.28 | 1.10 | 7.44 |
|  | Maximum | 27.00 | 40.40 | 19.00 | 30.20 | 93.00 | 17.40 | 2.14 | 13.30 |
|  | Minimum | 7.20 | 13.30 | 1.20 | 0.00 | 50.00 | 1.70 | 0.28 | 0.00 |
| **2022** | Average | 20.03 | 29.02 | 12.87 | 1.32 | 65.17 | 12.03 | 4.34 | 8.06 |
|  | Maximum | 30.80 | 44.00 | 21.10 | 23.20 | 98.00 | 17.90 | 7.60 | 13.30 |
|  | Minimum | 9.20 | 15.80 | -1.50 | 0.00 | 49.00 | -1.90 | 1.40 | 0.40 |
| **2023** | Average | 18.69 | 26.95 | 12.14 | 2.49 | 69.33 | 11.79 | 4.16 | 7.49 |
|  | Maximum | 31.00 | 42.50 | 21.30 | 52.80 | 99.00 | 20.50 | 10.60 | 13.30 |
|  | Minimum | 5.50 | 10.60 | -3.60 | 0.00 | 48.00 | -1.60 | 1.00 | 0.00 |
| **2024** | Average | 19.38 | 27.03 | 13.28 | 1.86 | 70.41 | 13.07 | 4.30 | 7.55 |
|  | Maximum | 29.40 | 41.00 | 22.20 | 25.50 | 96.00 | 20.30 | 7.80 | 13.30 |
|  | Minimum | 9.20 | 13.40 | 4.00 | 0.00 | 45.00 | 4.00 | 0.90 | 0.00 |

**Table S2.** Shapiro-Wilk statistics (W) and p-values for *Alternaria* spp. concentrations and average temperature (Avg T, °C), maximum temperature (Max T, °C), minimum temperature (Min T, °C), rainnfall (mm), relative humidity (RH, %), dew point (°C), wind speed (km/h), and hours of sunshine (h).

|  | Shapiro-Wilk normality test | |
| --- | --- | --- |
|  | W | p-value |
| *Alternaria* | 0.6597 | < 2.2E-16 |
| Avg T | 0.9972 | 0.1406 |
| Max T | 0.9930 | 0.0004 |
| Min T | 0.9877 | 1.19E-06 |
| Rainfall | 0.4185 | < 2.2E-16 |
| RH | 0.9625 | 3.98E-14 |
| Dew point | 0.9758 | 9.12E-11 |
| Wind speed | 0.9832 | 2.10E-08 |
| Sun hours | 0.9518 | 3.60E-16 |

**Table S3**. Coefficients of the generalized linear mixed models (GLMMs) with Tweedie distribution and log link used to assess the effects of meteorological variables and year on airborne *Alternaria* spp. concentrations. Each model includes a random intercept for Phenology stages. Fixed-effect estimates (Estimate), their standard errors (SE), z-values, and significance levels are presented. The variance and standard deviation of the random intercept are shown for each model. Akaike Information Criteria (AIC) and Bayesian Information Criteria (BIC) were used to select the best mixed generalized linear model.

|  |  | Estimate | Std. error | z - value | Pr(>\|z\|) |
| --- | --- | --- | --- | --- | --- |
| Model A | Intercept | 1.429 | 0.460 | 3.109 | 0.002 ** |
|  | Year 2022 | 0.383 | 0.114 | 3.370 | 0.001 *** |
|  | Year 2023 | 0.642 | 0.106 | 6.071 | 1.27E-09 *** |
|  | Year 2024 | 0.117 | 0.118 | 0.990 | 0.322 |
|  |  | Variance | Std.Dev. |  |  |
|  | Phenology | 0.813 | 0.902 |  |  |
|  | AIC | 3662.8 |  | BIC | 3693.7 |
| Model AT | Intercept | -0.547 | 0.396 | -1.379 | 0.168 |
|  | Year 2022 | 0.120 | 0.111 | 1.084 | 0.279 |
|  | Year 2023 | 0.466 | 0.102 | 4.556 | 5.22E-06 *** |
|  | Year 2024 | -0.186 | 0.116 | -1.602 | 0.109 |
|  | Avg T | 0.114 | 0.012 | 9.777 | < 2E-16 *** |
|  |  | Variance | Std.Dev. |  |  |
|  | Phenology | 0.433 | 0.658 |  |  |
|  | AIC | 3573.8 |  | BIC | 3609.1 |
| Model MT | Intercept | -0.626 | 0.423 | -1.479 | 0.139 |
|  | Year 2022 | 0.169 | 0.109 | 1.553 | 0.121 |
|  | Year 2023 | 0.536 | 0.101 | 5.329 | 9.89E-08 *** |
|  | Year 2024 | -0.059 | 0.112 | -0.527 | 0.598 |
|  | Max T | 0.079 | 0.008 | 10.370 | < 2E-16 *** |
|  |  | Variance | Std.Dev. |  |  |
|  | Phenology | 0.526 | 0.725 |  |  |
|  | AIC | 3563.1 |  | BIC | 3598.4 |
| Model MiT | Intercept | 0.797 | 0.415 | 1.921 | 0.055 |
|  | Year 2022 | 0.283 | 0.114 | 2.477 | 0.013 * |
|  | Year 2023 | 0.557 | 0.106 | 5.231 | 1.69E-07 *** |
|  | Year 2024 | -0.038 | 0.122 | -0.316 | 0.752 |
|  | Min T | 0.058 | 0.013 | 4.630 | 3.66E-06 *** |
|  |  | Variance | Std.Dev. |  |  |
|  | Phenology | 0.581 | 0.763 |  |  |
|  | AIC | 3643.5 |  | BIC | 3678.8 |
| Model R | Intercept | 1.507 | 0.463 | 3.258 | 0.001 ** |
|  | Year 2022 | 0.356 | 0.113 | 3.157 | 0.002 ** |
|  | Year 2023 | 0.632 | 0.105 | 6.037 | 1.57E-09 *** |
|  | Year 2024 | 0.087 | 0.117 | 0.741 | 0.459 |
|  | Rainfall | -0.029 | 0.008 | -3.462 | 0.001 *** |
|  |  | Variance | Std.Dev. |  |  |
|  | Phenology | 0.823 | 0.907 |  |  |
|  | AIC | 3652.2 |  | BIC | 3687.5 |
| Model RH | Intercept | 3.462 | 0.562 | 6.161 | 7.22e-10 *** |
|  | Year 2022 | 0.233 | 0.114 | 2.045 | 0.041 * |
|  | Year 2023 | 0.602 | 0.104 | 5.805 | 6.43E-09 *** |
|  | Year 2024 | 0.045 | 0.116 | 0.390 | 0.696 |
|  | RH | -0.028 | 0.004 | -6.273 | 3.53E-10 *** |
|  |  | Variance | Std.Dev. |  |  |
|  | Phenology | 0.816 | 0.904 |  |  |
|  | AIC | 3625.3 |  | BIC | 3660.6 |
| Model DP | Intercept | 0.616 | 0.422 | 1.458 | 0.145 |
|  | Year 2022 | 0.334 | 0.112 | 2.973 | 0.003 ** |
|  | Year 2023 | 0.565 | 0.106 | 5.333 | 9.67E-08 *** |
|  | Year 2024 | -0.032 | 0.121 | -0.268 | 0.789 |
|  | Dew Point | 0.072 | 0.015 | 4.902 | 9.47E-07 *** |
|  |  | Variance | Std.Dev. |  |  |
|  | Phenology | 0.571 | 0.756 |  |  |
|  | AIC | 3634.7 |  | BIC | 3670.0 |
| Model WS | Intercept | 1.389 | 0.471 | 2.951 | 0.003 ** |
|  | Year 2022 | 0.378 | 0.114 | 3.303 | 0.001 *** |
|  | Year 2023 | 0.640 | 0.106 | 6.033 | 1.61E-09 *** |
|  | Year 2024 | 0.114 | 0.120 | 0.948 | 0.343 |
|  | Wind Speed | 0.011 | 0.027 | 0.390 | 0.697 |
|  |  | Variance | Std.Dev. |  |  |
|  | Phenology | 0.812 | 0.901 |  |  |
|  | AIC | 3651.5 |  | BIC | 3686.7 |
| Model SH | Intercept | 0.781 | 0.455 | 1.718 | 0.086 |
|  | Year 2022 | 0.315 | 0.110 | 2.860 | 0.004 ** |
|  | Year 2023 | 0.571 | 0.102 | 5.575 | 2.48E-08 *** |
|  | Year 2024 | 0.015 | 0.115 | 0.131 | 0.896 |
|  | Sun Hours | 0.092 | 0.012 | 7.810 | 5.74E-15 *** |
|  |  | Variance | Std.Dev. |  |  |
|  | Phenology | 0.769 | 0.877 |  |  |
|  | AIC | 3598.6 |  | BIC | 3633.9 |
| Model ATMTSHRRH | Intercept | -1.085 | 0.724 | -1.498 | 0.134 |
|  | Year 2022 | 0.155 | 0.111 | 1.401 | 0.161 |
|  | Year 2023 | 0.487 | 0.102 | 4.777 | 1.78e-06 *** |
|  | Year 2024 | -0.144 | 0.118 | -1.226 | 0.220 |
|  | Avg T | 0.055 | 0.025 | 2.195 | 0.028 * |
|  | Max T | 0.035 | 0.019 | 1.825 | 0.068 |
|  | Rainfall | -0.013 | 0.009 | -1.444 | 0.149 |
|  | RH | 0.006 | 0.006 | 0.932 | 0.351 |
|  | Sun Hours | 0.037 | 0.018 | 1.999 | 0.046 * |
|  |  | Variance | Std.Dev. |  |  |
|  | Phenology | 0.492 | 0.701 |  |  |
|  | AIC | 3555.8 |  | BIC | 3608.8 |
| Model MTSHRRH | Intercept | -0.775 | 0.722 | -1.073 | 0.283 |
|  | Year 2022 | 0.185 | 0.110 | 1.677 | 0.094 . |
|  | Year 2023 | 0.531 | 0.100 | 5.301 | 1.15e-07 *** |
|  | Year 2024 | -0.066 | 0.112 | -0.587 | 0.557 |
|  | Max T | 0.069 | 0.010 | 6.700 | 2.08E-11 *** |
|  | Rainfall | -0.012 | 0.009 | -1.308 | 0.191 |
|  | RH | 0.004 | 0.006 | 0.553 | 0.580 |
|  | Sun Hours | 0.025 | 0.018 | 1.406 | 0.160 |
|  |  | Variance | Std.Dev. |  |  |
|  | Phenology | 0.553 | 0.744 |  |  |
|  | AIC | 3558.7 |  | BIC | 3607.2 |
| Model MTSHR | Intercept | -0.457 | 0.440 | -1.040 | 0.298 |
|  | Year 2022 | 0.174 | 0.109 | 1.605 | 0.109 |
|  | Year 2023 | 0.532 | 0.100 | 5.305 | 1.13E-07 *** |
|  | Year 2024 | -0.065 | 0.112 | -0.581 | 0.561 |
|  | Max T | 0.068 | 0.010 | 6.786 | 1.15E-11 *** |
|  | Rainfall | -0.010 | 0.008 | -1.195 | 0.196 |
|  | Sun Hours | 0.021 | 0.016 | 1.293 | 0.232 |
|  |  | Variance | Std.Dev. |  |  |
|  | Phenology | 0.561 | 0.749 |  |  |
|  | AIC | 3557.0 |  | BIC | 3601.1 |
| Model MTSHRH | Intercept | -0.582 | 0.708 | -0.822 | 0.411 |
|  | Year 2022 | 0.183 | 0.110 | 1.659 | 0.097 |
|  | Year 2023 | 0.531 | 0.100 | 5.293 | 1.20E-07 *** |
|  | Year 2024 | -0.061 | 0.113 | -0.540 | 0.589 |
|  | Max T | 0.068 | 0.010 | 6.578 | 4.78E-11 *** |
|  | RH | 0.001 | 0.006 | 0.126 | 0.900 |
|  | Sun Hours | 0.028 | 0.017 | 1.583 | 0.113 |
|  |  | Variance | Std.Dev. |  |  |
|  | Phenology | 0.555 | 0.745 |  |  |
|  | AIC | 3558.4 |  | BIC | 3602.5 |
| Model MTATSH | Intercept | -0.592 | 0.423 | -1.399 | 0.162 |
|  | Year 2022 | 0.147 | 0.110 | 1.336 | 0.182 |
|  | Year 2023 | 0.491 | 0.102 | 4.815 | 1.47E-06 *** |
|  | Year 2024 | -0.132 | 0.118 | -1.126 | 0.260 |
|  | Max T | 0.034 | 0.019 | 1.802 | 0.072 |
|  | Avg T | 0.051 | 0.025 | 2.055 | 0.040 * |
|  | Sun Hours | 0.035 | 0.016 | 2.231 | 0.026 * |
|  |  | Variance | Std.Dev. |  |  |
|  | Phenology | 0.504 | 0.710 |  |  |
|  | AIC | 3554.2 |  | BIC | 3598.3 |
| Model ATSH | Intercept | -0.471 | 0.415 | -1.136 | 0.256 |
|  | Year 2022 | 0.140 | 0.110 | 1.270 | 0.204 |
|  | Year 2023 | 0.466 | 0.101 | 4.605 | 4.12E-06 *** |
|  | Year 2024 | -0.175 | 0.115 | -1.520 | 0.128 |
|  | Avg T | 0.090 | 0.013 | 6.786 | 1.15e-11 *** |
|  | Sun Hours | 0.050 | 0.013 | 3.867 | 0.000 *** |
|  |  | Variance | Std.Dev. |  |  |
|  | Phenology | 0.494 | 0.703 |  |  |
|  | AIC | 3555.4 |  | BIC | 3595.1 |
| Model MTSH | Intercept | -0.512 | 0.436 | -1.173 | 0.241 |
|  | Year 2022 | 0.181 | 0.109 | 1.663 | 0.096 |
|  | Year 2023 | 0.531 | 0.100 | 5.295 | 1.19E-07 *** |
|  | Year 2024 | -0.061 | 0.113 | -0.540 | 0.589 |
|  | Max T | 0.067 | 0.010 | 6.732 | 1.68E-11 *** |
|  | Sun Hours | 0.027 | 0.015 | 1.753 | 0.080 |
|  |  | Variance | Std.Dev. |  |  |
|  | Phenology | 0.556 | 0.746 |  |  |
|  | AIC | 3556.4 |  | BIC | 3596.1 |
| Model RRH | Intercept | 3.350 | 0.579 | 5.785 | 7.26e-09 *** |
|  | Year 2022 | 0.236 | 0.114 | 2.071 | 0.038 * |
|  | Year 2023 | 0.602 | 0.104 | 5.809 | 6.30E-09 *** |
|  | Year 2024 | 0.042 | 0.116 | 0.364 | 0.716 |
|  | Rainfall | -0.007 | 0.009 | -5.245 | 1.56E-07 *** |
|  | RH | -0.026 | 0.005 | -0.804 | 0.421 |
|  |  | Variance | Std.Dev. |  |  |
|  | Phenology | 0.818 | 0.905 |  |  |
|  | AIC | 3626.6 |  | BIC | 3666.3 |
| Model ATDP | Intercept | -0.486 | 0.412 | -1.180 | 0.238 |
|  | Year 2022 | 0.103 | 0.111 | 0.923 | 0.356 |
|  | Year 2023 | 0.472 | 0.102 | 4.613 | 3.97E-06 *** |
|  | Year 2024 | -0.161 | 0.117 | -1.378 | 0.168 |
|  | Avg T | 0.132 | 0.016 | 8.436 | < 2E-16 *** |
|  | Dew Point | -0.032 | 0.019 | -1.690 | 0.091 |
|  |  | Variance | Std.Dev. |  |  |
|  | Phenology | 0.477 | 0.691 |  |  |
|  | AIC | 3567.5 |  | BIC | 3607.2 |
| Model MTDP | Intercept | -0.703 | 0.421 | -1.672 | 0.095 |
|  | Year 2022 | 0.168 | 0.109 | 1.544 | 0.123 |
|  | Year 2023 | 0.526 | 0.101 | 5.198 | 2.01E-07 *** |
|  | Year 2024 | -0.081 | 0.115 | -0.702 | 0.482 |
|  | Max T | 0.075 | 0.008 | 8.999 | < 2E-16 *** |
|  | Dew Point | 0.015 | 0.015 | 0.984 | 0.325 |
|  |  | Variance | Std.Dev. |  |  |
|  | Phenology | 0.491 | 0.701 |  |  |
|  | AIC | 3558.5 |  | BIC | 3598.2 |
| Model MTRH | Intercept | -0.230 | 0.670 | -0.344 | 0.731 |
|  | Year 2022 | 0.158 | 0.109 | 1.446 | 0.148 |
|  | Year 2023 | 0.536 | 0.101 | 5.329 | 9.87E-08 *** |
|  | Year 2024 | -0.060 | 0.112 | -0.536 | 0.592 |
|  | Max T | 0.075 | 0.009 | 8.062 | 7.49E-16 *** |
|  | RH | -0.004 | 0.005 | -0.766 | 0.444 |
|  |  | Variance | Std.Dev. |  |  |
|  | Phenology | 0.541 | 0.736 |  |  |
|  | AIC | 3564.5 |  | BIC | 3604.2 |
| Model MiTDP | Intercept | 0.621 | 0.419 | 1.482 | 0.138 |
|  | Year 2022 | 0.313 | 0.115 | 2.722 | 0.006 ** |
|  | Year 2023 | 0.557 | 0.106 | 5.235 | 1.65E-07 *** |
|  | Year 2024 | -0.044 | 0.122 | -0.365 | 0.715 |
|  | Min T | 0.020 | 0.023 | 0.859 | 0.390 |
|  | Dew Point | 0.052 | 0.027 | 1.922 | 0.055 |
|  |  | Variance | Std.Dev. |  |  |
|  | Phenology | 0.559 | 0.748 |  |  |
|  | AIC | 3636.0 |  | BIC | 3675.7 |

Average temperature (Avg T, °C), maximum temperature (Max T, °C), minimum temperature (Min T, °C), rainfall (mm), relative humidity (RH, %), dew point (°C), wind speed (km/h) and sun hours (h). Significance codes: 0 ‘***’ 0.001 ‘**’ 0.01 ‘*’.
